# Extremely Rare Presentation of Pilonidal Sinus Disease in the Posterior Cranial Fossa of a 2-Year-Old Patient: A Case Report

**DOI:** 10.1055/a-2641-6301

**Published:** 2025-07-11

**Authors:** Hamzeh Yacoub, Aya Aqel, Mohammed Adas, Qais Hjouj, Zaid Yacoub, Rita Yacoub, Hadi Dababseh

**Affiliations:** 1 Faculty of Medicine, Al-Quds University, Jerusalem, Palestine; 2 Neurosurgery Department, Istishari Arab Hospital, Ramallah, Palestine

**Keywords:** pilonidal sinus, cranial cystic lesion, posterior cranial fossa, pediatric neurosurgery

## Abstract

A 2-year-old female patient presented after experiencing a generalized tonic-clonic seizure accompanied by fever, followed by a loss of consciousness. She underwent an urgent right frontal external ventricular drain placement. Intraoperative cerebrospinal fluid analysis was negative for infectious patterns. MRI showed a predominantly cystic lesion in the midline posterior fossa, with a compressive mass effect. Subsequently, she underwent a suboccipital craniotomy for microscopic resection of a posterior cranial fossa lesion. Histopathology reported keratin flakes with severe active inflammation, and foreign body type giant cell reaction in scalp excision with free hair shafts through the inflammatory focus, consistent with pilonidal sinus. The patient was then discharged home in good health.

## Introduction

Pilonidal sinus (PNS) is a deep cyst with single or multiple tracts, characterized by the presence of pus and loose hair utilizing the space. 1 While classically occurring in the sacrococcygeal region, 1 rare cases have been reported in atypical locations such as the scalp, neck, and axilla. However, abdominal and penile lesions are the least common. 2 3 The dominant theory behind its occurrence was first believed to be congenital, owing to the absence of the primitive ectoderm. 1 Years later, the Acquired Theory has emerged, linking the cause to the microtraumatization by the hair follicles. A Pilonidal cyst is generally asymptomatic until it becomes inflamed, forming a pilonidal abscess. 4 It is twice as frequent in men as in women. Additionally, the age at presentation is 21 years for men, and 19 years for women. 5

Treatment of PNS is usually not indicated unless the PNS is infected, which then requires a surgical procedure followed by antibiotics and painkillers. 4

Herein, we report the first documented case of PNS located in the posterior cranial fossa of a pediatric patient.

## Clinical Presentation

A 2-year-old girl was referred to the pediatric neurosurgery department of our hospital due to a suspicious posterior cranial fossa mass. The patient has a prior history of meningitis at 40 days and 4 months of age, with a free surgical history. One month before admission, the patient began experiencing increased sleeping hours, hypoactivity throughout the day, daily vomiting, and an unbalanced gait. Additionally, she had a localized posterior headache. Two weeks ago, she developed an intermittent fever, which measured 38°C, for which she was treated as a case of upper respiratory tract infection with antibiotics and antipyretics; the fever responded to medications until discontinuation. Upon admission, she experienced a generalized tonic-clonic seizure accompanied by fever, followed by a loss of consciousness. She was suspected of having meningitis until she underwent a computed tomography (CT) scan, which revealed a huge mass with obstructive hydrocephalus features.

On physical examination, the patient appeared ill, with a decreased level of consciousness and no response to verbal or physical stimuli. Inspection of the head revealed mild erythema over the suboccipital area but no evident masses or discharge. Her head circumference was 47 cm, height 84 cm, and weight 10 kg. Notably, the PNS tract was not observed during the initial inspection. In retrospect, the tract may have been missed due to its small punctate nature and the presence of dense hair in the suboccipital region, which likely obscured the opening.

On the day of admission, the patient underwent an urgent right frontal external ventricular drain (EVD) placement at the level of Kocher's point. Intraoperative cerebrospinal fluid (CSF) analysis showed no signs of infection (WBCs: 0, RBCs: 0, glucose: 116 mg/dL, and proteins: 5.6 mg/dL).

**Fig. 1:**
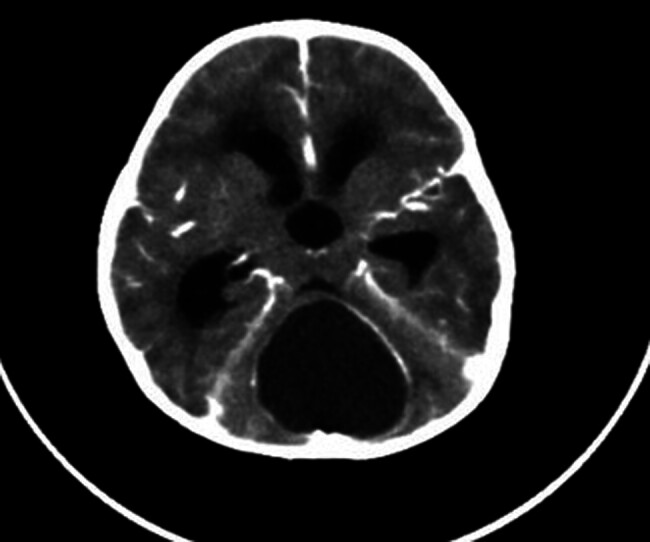
A CT scan of the cranium demonstrates the posterior cranial fossa mass after presentation.

**Fig. 2:**
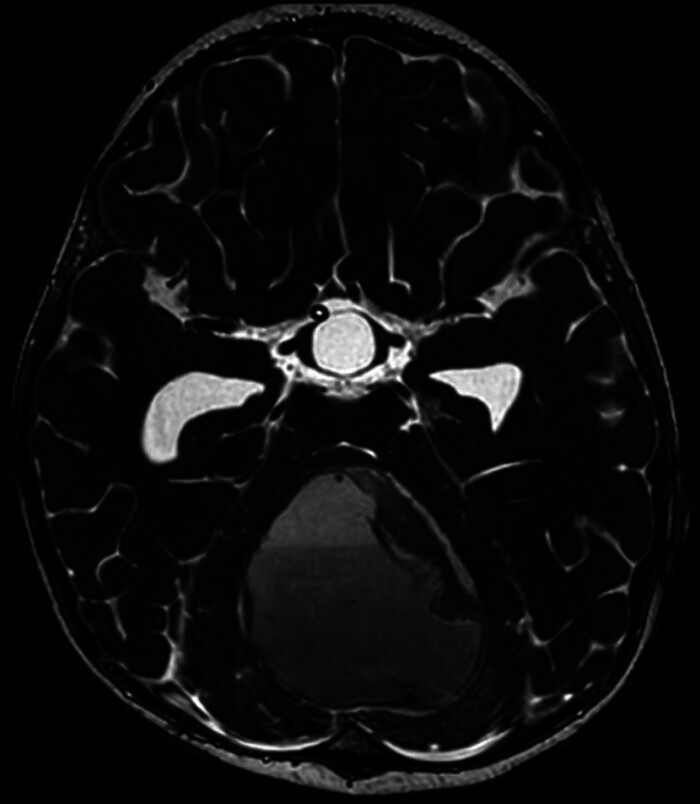
MRI demonstrating a large cystic mass in the posterior cranial fossa.

**Fig. 3:**
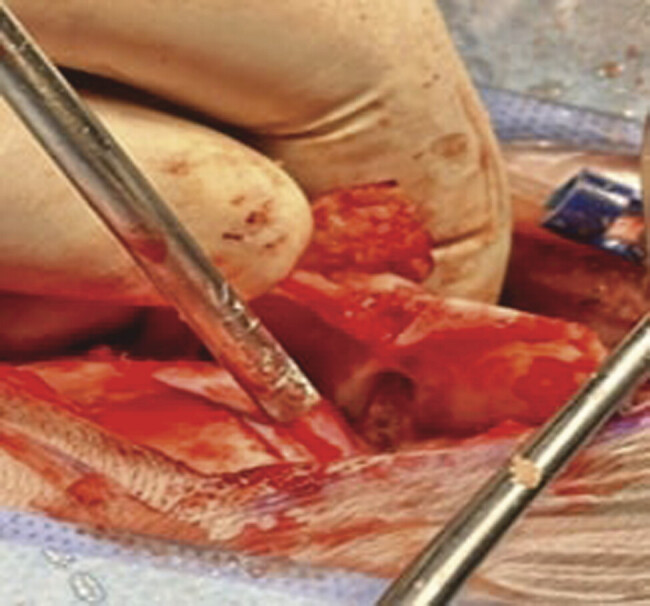
Intraoperative, demonstrating the sinus between the scalp and the pilonidal cyst.

**Fig. 4:**
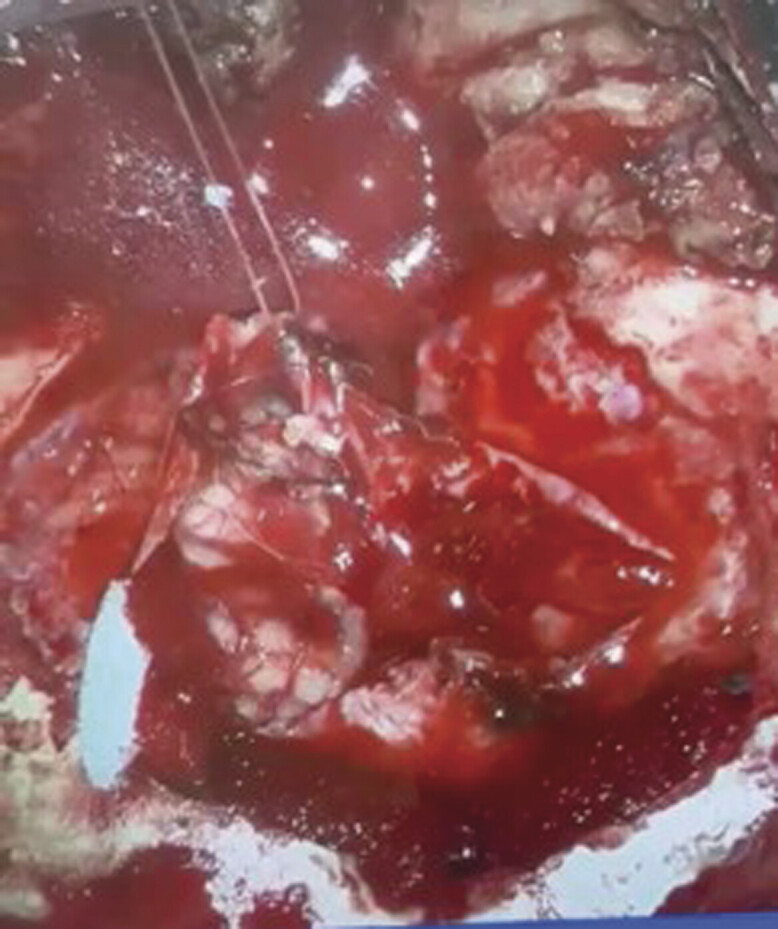
Intraoperative, demonstrating pus and keratinized tissue, and hair shafts.

After stabilization, CT scan was done and showed a midline posterior fossa, predominantly a cystic lesion with compressive mass effect, located at the anatomic location of the vermis ( Fig. 1 ). MRI was also performed and confirmed the cystic nature and location of the lesion ( Fig. 2 ). The morphology and the radiological appearance directed the differential diagnosis toward Juvenile Polycystic Astrocytoma or brain abscess. The patient underwent a suboccipital craniotomy for microscopic resection of a posterior cranial fossa lesion. Skin incision and bone release were done. Two Burr Holes were made, followed by suboccipital craniotomy with adequate exposure of the sinuses and foramen magnum. A Sinus was seen reaching the dural layer, causing a fistula. The midline approach was done with release of adhesions, then a gush of pus and hair was seen protruding out. A sample was taken for culture. Resected bone, sinus, and scalp were sent to histopathology. Gross examination revealed a big cerebellar mass composed of keratinized tissue and hair ( Figs. 3 and 4 ).

**Fig. 5:**
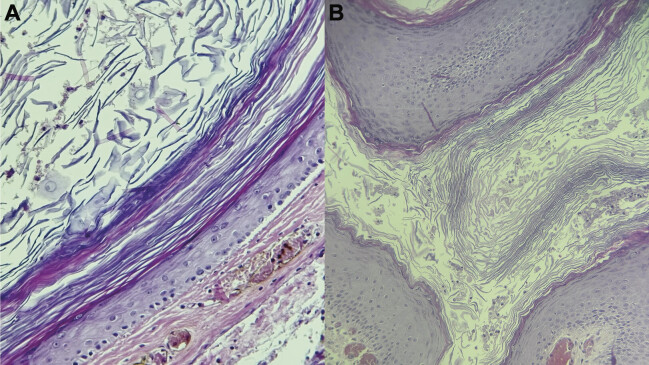
( **A** , **B** ) Microscopic exam reveals a dermal cyst lined by stratified squamous epithelium. Lamellated keratinous material is present in the cyst lumen.

**Fig. 6:**
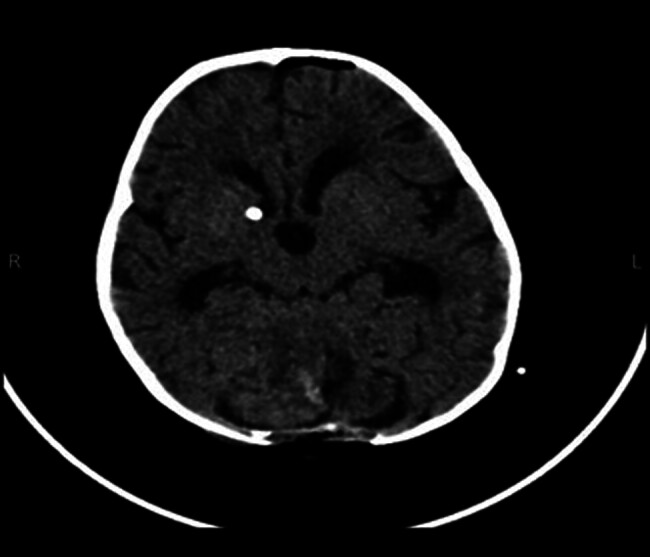
Postoperative CT scan.

Postoperatively, the patient was admitted to the pediatric intensive care unit. The patient recovered with no complications. The limb muscle strength was normal, with no ataxia or other abnormalities. Histopathology reported keratin flakes with severe active inflammation, negative for malignancy. It also found foreign body type giant cell reaction in scalp excision with free hair shafts through the inflammatory focus consistent with PNS ( Fig. 5 ). Postoperative CT scan showed normal cerebral parenchyma and CSF spaces, and no midline shift. Postoperative changes such as edema, hemorrhagic foci, and pneumocephalus were seen at the posterior fossa ( Fig. 6 ). Her postoperative laboratory results were normal, and no signs of fever were present. Four days after her surgery, she was doing well and active; she was discharged with no complications.

## Discussion

This case represents the first report of PNS in the pediatric posterior cranial fossa. This location is unique among all reported pilonidal diseases of the scalp. PNS is known to be very rare in the reported location and age group, and is also less common in females sex. 6

PNS classically presents in the sacrococcygeal region, 7 however, rare cases have showed atypical sites, notably the cheek, 8 axilla, 9 penis, 10 11 12 umbilicus, 13 periungual region, 14 neck, 2 15 postauricular, 16 17 intermammary, 18 and endoanal canal. 19 20 Noteworthy, most reported cases demonstrated male predominance in PNS occurrence, and were primarily seen in adults, which highlights the rarity of our case involving a female child. Table 1 summarizes the sites of some reported PNS.

**Table 1:** Overview of some sites of reported PNS

Patient	Age (y)	Gender	Lesion site
1	37	Male	Cheek 8
2	28	Male	Penis 10
3	27	Male	Axilla 9
4	28	Male	Postauricular 16
5	50	Female	Periungual 14
6	21	Male	Umbilicus 13
7	31	Male	Endoanal 20

PNS is usually asymptomatic until it becomes inflamed, hence it usually presents as a case of inflammation. 4 This patient presented to the hospital complaining of generalized tonic-clonic seizure accompanied by fever, localized posterior headache, unbalanced gait, vomiting, loss of consciousness, and nonspecific symptoms like hypoactivity and increased sleeping. Also, she had recurrent meningitis with a free surgical history; lastly, before admission by 2 weeks, she had an upper respiratory infection. CSF Analysis was done to make the diagnosis, as meningitis was suspected. However, neuroimaging preceded the lumbar puncture (LP), in accordance with the criteria for neuroimaging (CT\MRI) before LP in suspected meningitis, which include focal neurological deficit, altered mental status, immunocompromised state, increased intracranial pressure, and new onset seizure. 21

On brain CT scan, a posterior fossa with a well-defined hypodense mass (4.3 × 4.3) cm with obstructive hydrocephalus (ventriculomegaly) due to mass effects was observed ( Fig. 1 ). Acute obstructive hydrocephalus is a medical emergency. Managed by the placement of an EVD (shunt) to divert excess CSF from the ventricles to a body cavity where it is absorbed into the systemic circulation. 22

Accordingly, the shunt was inserted at the right frontal lateral ventricle smoothly without any complications.

Brain MRI with IV contrast showed a midline posterior fossa mass, predominantly cystic, lesion with compressive mass effect, located at the anatomical location of the vermis. The morphology and radiological appearances, particularly the wall, the fluid–fluid level, and the diffusion restriction, direct the differential diagnosis toward brain abscess ( Fig. 2 ).

However, other differentials, including complicated cystic-neoplastic masses, like juvenile pilocytic astrocytoma still in the differentials. Astrocytoma is less likely because it is a slow-growing tumor, and the size of the lesion, according to the patient's age, doesn't correlate. 23

Definitive diagnosis was done through the pathology lab after lesion resection by posterior fossa craniotomy. Intraoperatively, a sinus was seen between the scalp and the pilonidal cyst, causing a fistula ( Fig. 3 ). Interestingly, despite the clear intraoperative findings, the lesion was not detected during the initial physical exam. The initial physical examination failed to reveal the PNS tract. This may be attributed to its small size and the dense hair growth in the suboccipital region, which obscured the punctate opening. This highlights the diagnostic difficulty in rare cranial presentations of pilonidal disease and raises the possibility of underdiagnosis in similar cases.

## Conclusion

Although PNS is highly rare in the posterior cranial fossa, it should be taken into consideration in a posterior cranial mass of a pediatric age group when hair is seen inside the cyst cavity. These lesions, which are easy to control and treat when diagnosed correctly, can rarely turn into malignancy when they remain untreated for a long period or when incomplete treatment is administered. Thus, more literature information about PNS in the head and neck region is needed.
